# Two-dimensional metal-organic frameworks: from synthesis to bioapplications

**DOI:** 10.1186/s12951-022-01395-9

**Published:** 2022-05-02

**Authors:** Weiqi Wang, Yuting Yu, Yilan Jin, Xiao Liu, Min Shang, Xiaohua Zheng, Tingting Liu, Zhigang Xie

**Affiliations:** 1 School of Pharmacy, Nantong University, Nantong, 226001 Jiangsu Province China; 2 Department of Medical Imaging, Affiliated Hospital of Nantong University, Nantong, 226001 Jiangsu Province China; 3 State Key Laboratory of Polymer Physics and Chemistry, Changchun Institute of Applied Chemistry, Chinese Academy of Sciences, Changchun, 130022 China

**Keywords:** Metal-organic framework, Two-dimensional materials, Diagnostic, Biosensing, Cancer treatment

## Abstract

As a typical class of crystalline porous materials, metal–organic framework possesses unique features including versatile functionality, structural and compositional tunability. After being reduced to two-dimension, ultrathin metal-organic framework layers possess more external excellent properties favoring various technological applications. In this review article, the unique structural properties of the ultrathin metal-organic framework nanosheets benefiting from the planar topography were highlighted, involving light transmittance, and electrical conductivity. Moreover, the design strategy and versatile fabrication methodology were summarized covering discussions on their applicability and accessibility, especially for porphyritic metal-organic framework nanosheet. The current achievements in the bioapplications of two-dimensional metal-organic frameworks were presented comprising biocatalysis, biosensor, and theranostic, with an emphasis on reactive oxygen species-based nanomedicine for oncology treatment. Furthermore, current challenges confronting the utilization of two-dimensional metal-organic frameworks and future opportunities in emerging research frontiers were presented.

**Graphical Abstract:**

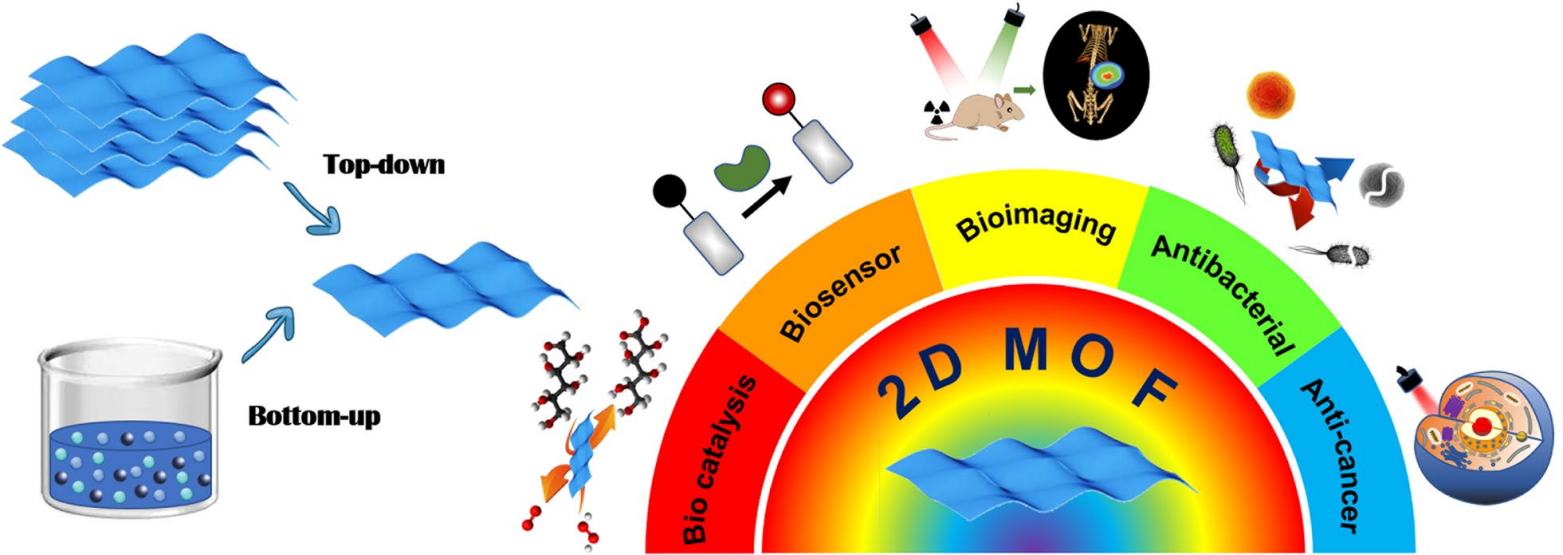

## Introduction

As a rapidly developing class of hybrid materials, metal-organic frameworks (MOFs) [1] constructed by metal–ligand coordination into diverse supramolecular architectures encompass different dimensions. Compared to organic or inorganic materials, one typical advantage of well-defined supramolecular architectures is the tunable structure through nearly infinite metal ions or clusters bridged by polydentate organic linkers. Except for the compositional versatility, MOFs exhibit high surface-to-volume ratios, various topology, and flexible porosity. Moreover, the functionalization of MOFs would be easily obtained either by modification of the building blocks previously or post-modification of pre-synthesized MOFs. Owing to these outstanding features, MOFs have been widely applied in the field of gas storage [2] and separations [3, 4], nonlinear optics [5], catalysis [6, 7], and sensing [8, 9].

By choosing biologically friendly compositions, MOFs with good biocompatibility have been extensively investigated in the areas across biology and nanomedicine [10, 11]. For biomedical applications, the infinite array built from metal–ligand coordination endows the inherent biodegradability of MOFs under physiological conditions. Besides, the flexible porosity confers MOFs as candidates of nanocarriers for loading different types of active ingredients involving small molecules, peptides/proteins, or nucleic acids with high loading capacity [12, 13]. Furthermore, the unique optical and/or X-ray attenuation abilities of MOFs can be harnessed for molecular imaging, including fluorescence imaging, computed tomography [14], magnetic resonance imaging [15], and positron emission tomography [16]. For therapeutic applications, especially for phototherapy or radiotherapy, photosensitizers and high-Z elements acting as organizing molecular building blocks integrated into the MOFs will augment reactive oxygen species (ROS) production [17, 18]. This desirable performance result from the enhanced intersystem crossing process and avoided aggregation of hydrophobic photosensitizers simultaneously. The two-dimensional MOFs (2D MOFs) are usually comprised of multiple ultrathin layers, possessing the characteristics of easy exposure of active sites and large surface area, which will embrace the great potential for various technological applications [19].

Similar to other 2D nanomaterials, the planar topography endows materials the intriguing physicochemical properties, including ductility, light transmittance, and electrical conductivity [20, 21]. Since the discovery of graphene in 2004 [22, 23], nanomaterials with high ratios of lateral size to thickness have been widely explored in biomedical applications. Up to date, 2D nanomaterials including transition metal dichalcogenides [24, 25], metal carbonitrides [26, 27], black phosphorus nanosheets [28, 29], layered double hydroxides [30, 31], MOFs, and covalent-organic frameworks[32] have aroused tremendous research attention in both synthetic methodology and/or practical applications. In particular, 2D MOFs displayed tailored structure, adjustable composition, efficient cargo loading, and good biodegradation, making these layered materials useful for biomedical applications [33]. Compared to the pristine MOFs, the ultrathin -nanosheet structures of 2D MOFs show distinct advantages for theranostic applications because of large surface areas, unique surface chemistry, and inherent optical properties derived from the fascinating topology. In particular, MOFs with ultrathin layered structures allow for rapid diffusion of ROS, resulting in the elevated therapeutic performance of ROS-based treatment modalities.

**Scheme 1:**
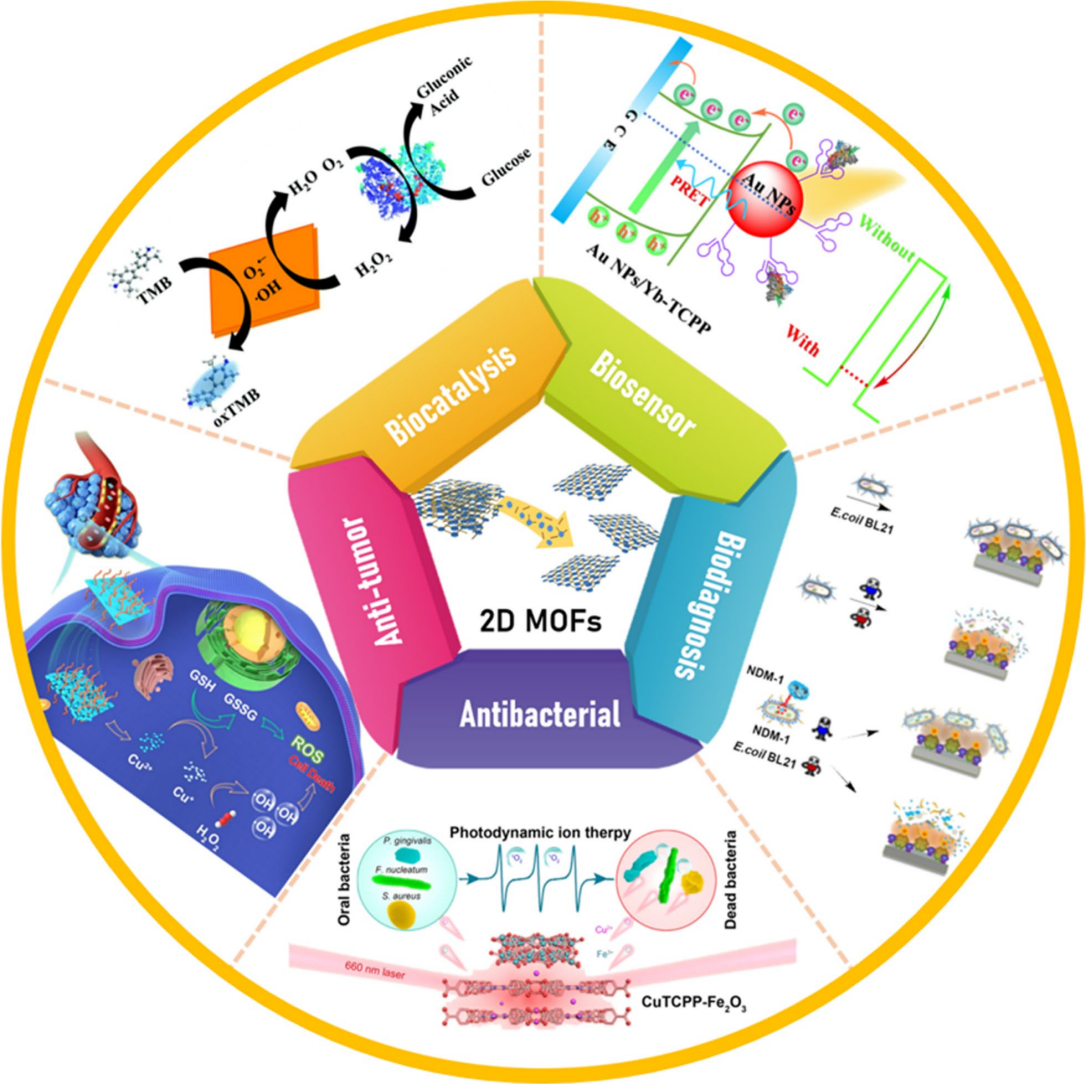
Graphical representation of the rational design of ultrathin MOF nanosheets and their application for biocatalysis and biosensor, disease diagnosis, molecular imaging, cancer therapy, and anti-bacterial/anti-infection properties. Reproduced with permission [36]. Copyright 2022, ACS publications. Reproduced with permission [37]. Copyright 2020, RSC publications. Reproduced with permission [38]. Copyright 2021, ACS publications. Reproduced with permission [39]. Copyright 2021, ACS publications. Reproduced with permission [40]. Copyright 2021, ACS publications. Reproduced with permission [41]. Copyright 2022, Springer Nature

So far, several systematic reviews have overviewed the synthetic methodology comprising the dimensional transformation, and their application in energy storage and/or functional devices [34, 35]. Herein, we systematically summarized the latest advances of 2D MOFs applied in bio-related applications, especially for cancer theranostic applications (Scheme 1) [36–41]. First, we summarized the synthesis and surface modification strategies of various types of 2D MOFs, focusing on structure–property relations. Then, we overviewed the most recent progress of multifunctional 2D MOFs in biomedical applications, including biocatalysis and biosensor, disease diagnosis, molecular imaging, malignancy management, and regenerative medicine. Emphasis is placed on the ROS-based treatment, involving photodynamic therapy (PDT) [42, 43], radioisotope therapy [44, 45], radiodynamic therapy (RDT) [46], chemodynamic therapy (CDT) [47, 48], and ROS-synergized cancer therapy. Finally, the existing challenges and outlooks in 2D MOFs are proposed based on the current development status and future requirements.

### Synthetic methodology

The design strategy is of significant importance to control the dimensionality of nanomaterials. So far, the synthetic routes containing mechanical cleavage, liquid exfoliation, chemical vapor deposition, and wet chemistry, have been widely investigated [49–52]. Generally, strategies to construct 2DMOF materials have been classified into top-down and bottom-up approaches (Table 1) [53–60]. In the top-down strategy, namely the dimensional reduction strategy, harsh mechanical or chemical conditions are required for exfoliating the bulk materials into ultrathin nanosheets, such as mechanical shaking, ball milling, ultrasonic treatment, and insertion stripping [61]. Compared with the top-down method, the bottom-up approach is more flexible and designable to synthesize ultrathin nanomaterials by utilizing appropriate precursors. The bottom-up strategy, also known as bulk solution preparation, comprises interface synthesis, surfactant-assisted synthesis, and modulated synthesis, which has the advantages of easy control over the growth direction with a high yield.

**Table 1:** The constituent composition, synthetic methodology, and physicochemical properties of porphyritic MOF nanosheets

Materials	2D MOFs		Methodology	Thickness (nm)	Refs.
	Metal	Ligand			
Zn_2_(PdTCPP)	Zn(NO_3_)_2_·6H_2_O		Intercalation and chemical exfoliation	~ 1	[53]
UNs-FA	ZrCl_4_		Solvothermal reaction	1.48 ± 0.22	[54]
Zn-TCPP(Fe)	Zn^2+^		Surface-assisted approach	1–1.5	[55]
Cu-TCPP(Fe)	Cu(NO_3_)_2_·3H_2_O		Surfactant-assisted approach	3.5 ± 1.2	[56]
M-TCPP(Fe)	Zn^2+^; Co^2+^; Cu^2+^		Surfactant-assisted approach	–	[57]
PPF-Gd NS	Gd^3+^		Surfactant-assisted approach	21.0 ± 9.4	[58]
Fe-TBP	[Fe_3_O(OAc)_6_(H_2_O)_3_]OAc		Solvothermal reaction	–	[59]
Hf-MOL	Hf_12_(m_3_-O)_8_(m_3_-OH)_8_ (m_2_-OH)_6_		Solvothermal reaction	1.6	[60]

Similar to the traditional synthetic strategy of 2D materials, several MOFs are suitable for exfoliation into ultrathin nanosheets while maintaining their crystallinity. So far, ultrasonic exfoliation is a general strategy for the exfoliation of bulk MOFs into MOF nanosheets by breaking the weak interactions between adjacent layers. For chemical exfoliation, pioneered work reported by Zhou et al. exploited chemically labile intercalating agent 4,4′-dipyridyl disulfide and trimethylphosphine to exfoliate crystalline MOFs (Zn2(PdTCPP)) built from tetrakis(4-carboxyphenyl)porphyrin (TCPP) and Zn^2+^ to achieve single-layer nanosheets [53]. These nanofabrication techniques result in 2D MOFs with a highly uniform size distribution, however, MOF nanosheets isolated by exfoliation require harsh mechanical or chemical conditions. Meanwhile, the top-down exfoliation process requires extensive optimization and precise control, which usually leads to an extremely low yield. As a comparison, the direct assembly of metal ions and organic linkers through the bottom-up approach to fabricate MOF nanosheets is more convenient. With the accessibility to the planar structure, the surfactant-assisted synthesis method gaining control over the crystallization process has shown great potential for practical application. As a paradigm, surfactant-assisted pre-organization of the metallic precursor was developed to synthesize NH_2_-MIL-53(Al) nanosheets, which is favored by the formation of oligomeric structures [62]. These MOF nanosheets exhibit a superior performance over other crystal morphologies in both chemical sensing and gas separation. In addition, exploiting monocarboxylic acids as “modulators”, He et al. have developed atomic thickness zirconium-porphyrinic MOF nanosheets with high-yield through one-pot solvothermal reaction [54]. Due to the planar topography, the 2D porphyrinic MOFs exhibited excellent-singlet oxygen generation capability and superior photocatalytic activity.

Apart from general synthetic considerations for 2D nanomaterials, engineering 2D MOFs synthetic routines involve a careful selection of organic spacer linkers with appropriate bridging groups to coordinate with metal ions/clusters with specific directionality [63]. Alternatively, the self-assembly of organic ligands and metal nodes enables the formation of MOFs with an atomic-layered structure during solution processing. To maximize the advantages of MOF nanosheets, the design strategy for the 2D MOFs is also concerned with the metal–ligand ratio, coordination number, coordination geometry, coordination environment, and structural transformation. Despite green synthesis and modification, the fabrication of large-scale 2D MOFs with high quality still faces huge challenges. The facile synthetic methodology is highly urgent to make these atomically ordered networks.

### Bioapplications of 2D MOFs

Advancements in 2D MOFs for biomedical applications have occurred at a relatively slow pace compared with other 2D nanomaterials. With the diversity in the structural phases and the unique properties, the atomic-layered 2D MOFs have extraordinary advantages when exploited for various bioapplications [63, 64]. This section focused on the bioapplications of 2D MOFs for biocatalysis and biosensor, biological imaging, and theranostics.

### Biocatalysis and biosensor

The selectivity towards target molecules is generally affected by the choice of the organic linkers and/or the metal ions that are introduced into the MOF structures. For example, different metal ions are usually accompanied by differentiated properties, such as magnetism, photoluminescence, as well as catalytic or sensing activity. As a comparison, MOF with sheet-like morphology confers their structure allows for more active site exposure for site-specific biocatalysis and biorecognition with higher sensitivity. Concerning bioapplications, 2D MOFs have the potential to provide an efficient solution to the detection of heavy metals, medications, proteins, and exosomes (Table 2) [38, 65–69].

**Table 2:** Representative porphyritic MOF nanosheets are used for the detection of biomarkers

2D MOFs	Biomarkers	Limitations	Sources	Refs.
Au NPs/Yb-TCPP	SARs-CoV2 spike glycoprotein	72 ng/mL	Throat swabs	[38]
Cu-TCPP(Fe)	Protein	0.742 pg/mL	Serum	[65]
Cu-TCPP	PD-L1 exosomes	16.7 particles/mL	Human serum	[66]
Cu-HHTP	Chemiresistive NH_3_	0.5 ppm	–	[67]
Yb-MOF	Picric acid and berberine chloride form	81.3 nM/36.5 nM	–	[68]
Cu-TCPP	Lead ions	3.3 nM	Tap water and fertilizer	[69]

**Fig. 1:**
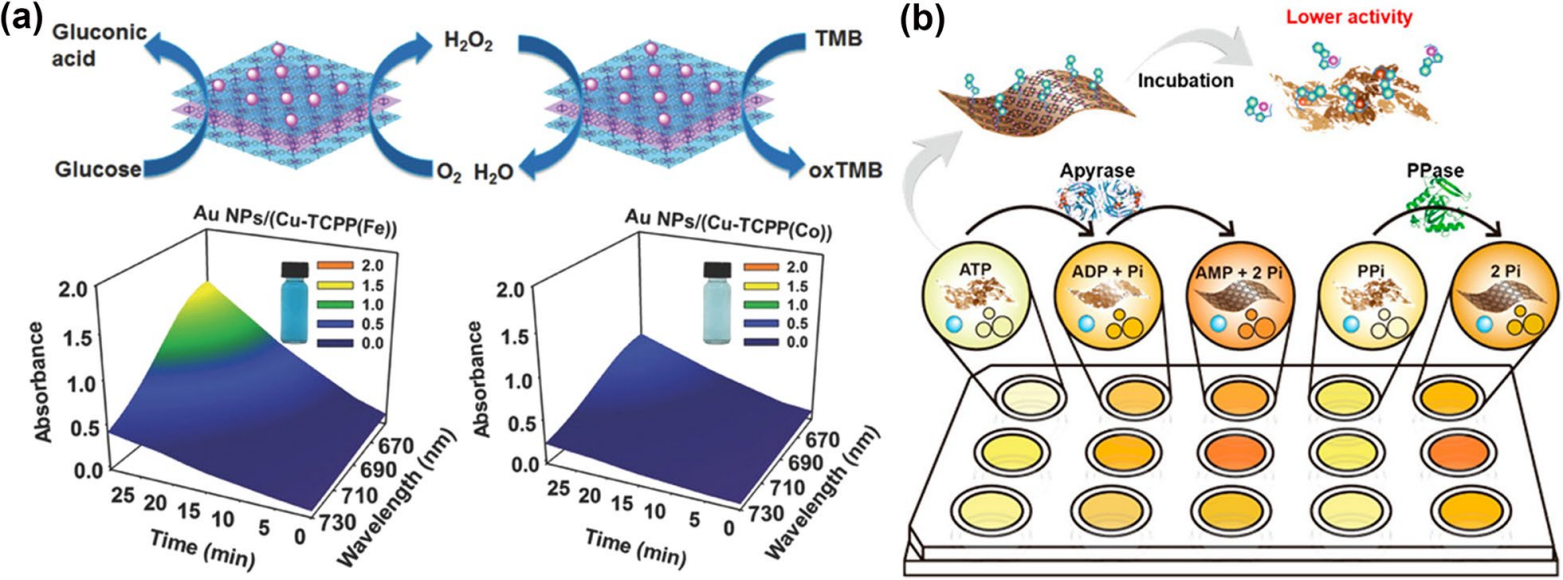
**a** Graphical representation of the enzyme-mimetic cascade reaction and absorption spectra after Au NPs/Cu-TCPP(Fe) or Au NPs/Cu-TCPP(Co) catalysis. Reprinted with permission from Ref. [56], Copyright 2017, Wiley–VCH. **b** Ultrathin metalloporphyrins act as sensor arrays to monitor various phosphates. Reprinted from Ref. [57] with permission of American Chemical Society, Copyright 2018

As an emerging nanoenzyme, MOFs with tunable structures and robust stability have aroused wide attention. Previous studies revealed that metalloporphyrins-based MOFs with layered topology exhibited an augmented peroxidase-mimicking activity when compared to their bulk analogs [67]. Employing the layered structure, Huang et al. designed and developed a nanoreactor (Au NPs/Cu-TCPP(Fe) or Au NPs/Cu-TCPP(Co)) to mimic the enzymatic cascade reactions [56]. A nano-biomimetic system was constructed by incorporating Au nanoparticles around 2.1 ± 0.5 nm onto the layered MOFs pre-synthesized by the surface-assisted method. In this biomimetic system, ultrasmall Au nanoparticles acting as glucose oxidase together with metalloporphyrins MOF nanosheets mimicking peroxidase facilitated the oxidation of 3,3′,5,5′-tetramethylbenzidine and glucose simultaneously (Fig. 1a). As a proof of concept study, the absorbance at 652 nm for oxidation of 3,3′,5,5′-tetramethylbenzidine and the glucose concentrations displayed linear relationship ranges from 10 × 10^–6^ M to 300 × 10^–6^ M, and the colorimetric result can be identified by the naked eyes directly. More importantly, this study may open up a new avenue to design metalloporphyrins MOF-based nanosheets enabling biological catalysts with high catalytic activity, substrate specificity, and sustainability.

Due to the modular nature and unique surface, the layered MOFs with atomically precise structures have emerged as the prospective nanoplatforms for efficient catalysts within bioinspired systems. Given that, numerous approaches have been devoted to fabricating MOFs with a planar topography and the superior characteristics toward biocatalysis applications, including structural flexibility, required porosity, and inherent optical properties. Besides, MOFs with layered morphology is beneficial for minimizing the diffused barriers and maximizing the catalytic activity. The top-down synthesized MOF nanosheets were built from copper trifluoromethanesulfonate and 4,4-bipyridine [70]. The detection of hydrogen peroxide and glucose was relied on converting non-fluorescent thiamine to strong fluorescent thiochrome in the presence of hydrogen peroxide. The selectivity and sensitivity of the biosensor were augmented when combined with glucose oxidase because of the supplement of hydrogen peroxide. The as-designed fluorescent biosensor with a low detection limit and wide ranges are established for clinical diagnosis indicators of glucose concentration in human serum. Mimicking the peroxidase, ultrathin metalloporphyrins MOFs were utilized to construct sensor arrays for the detection of phosphates and their enzymatic hydrolysis (Fig. 1b) [57]. Intriguingly, the selectivity of sensor arrays is originated from adjustable peroxidase-mimicking activity, which can give cross-reactive signals to discriminate multiple phosphates simultaneously. This work demonstrates a MOF-based nanoenzyme with tunable enzyme activity, providing a convenient and reliable analytical platform for practical application in biological samples.

### Biodiagnosis

**Fig. 2:**
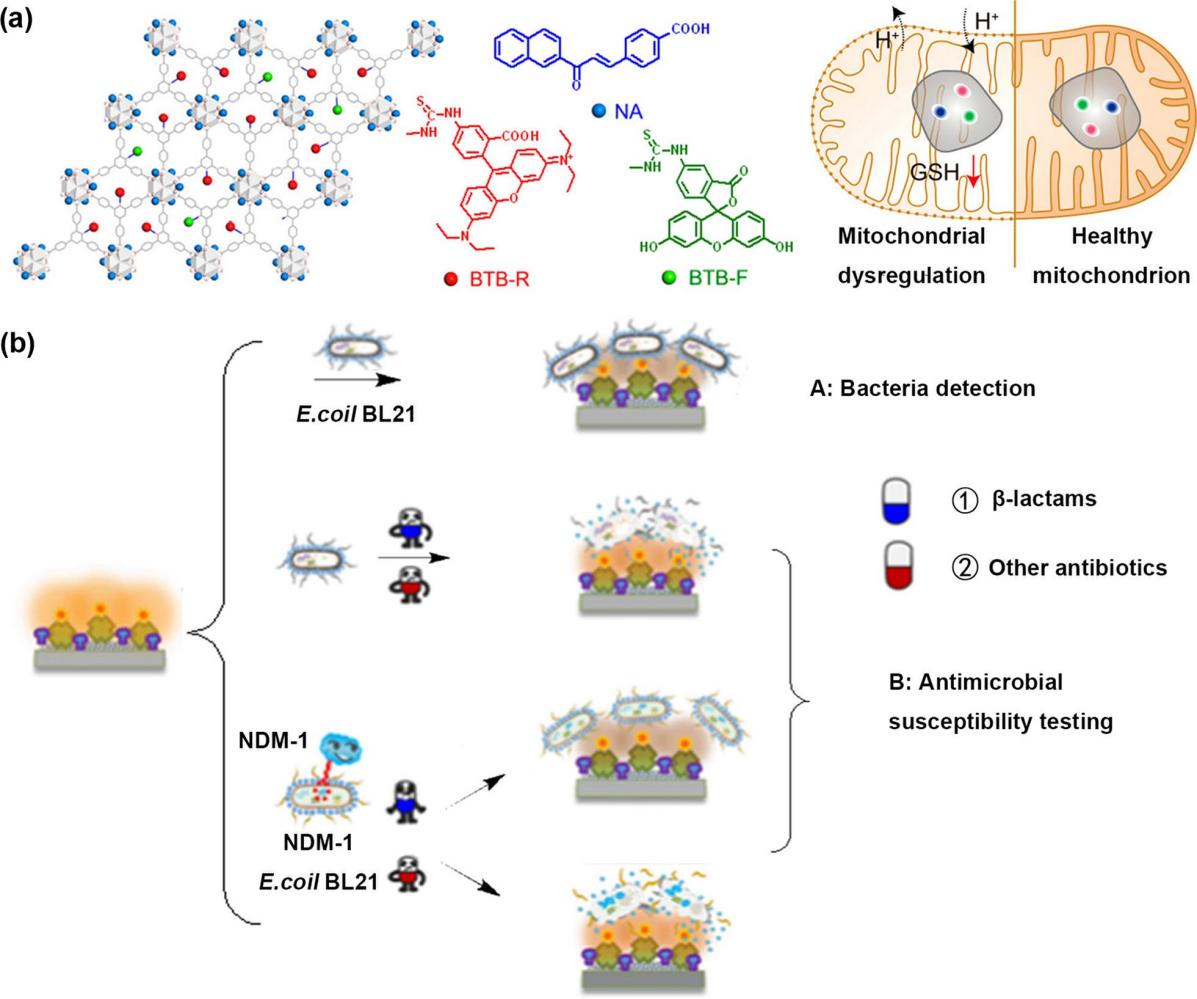
**a** Schematic illustration of the utilization of NA@Zr-BTB/F/R to ratiometrically sense dysregulated mitochondria with GSH depletion and matrix acidification. Reprinted from Ref. [73] with permission of American Chemical Society, Copyright 2021. **b** Representation of Ru-Con A@ NH_2_-MIL-53(Al) for the detection of Gram-negative bacteria and antimicrobial susceptibility through electrochemiluminescence. Reprinted from Ref. [39] with permission of American Chemical Society, Copyright 2021

Compared to the bulk MOF crystals, 2D MOFs possess enhanced performance in catalysis and sensing application. These advantages are partly due to the ultrathin layer structures, which are capable of facilitating the exposure of the active site and the diffusion of the reactive matrix. Thereby, 2D MOFs with increased specificity towards biological entities have been employed for biological detections comprising glucose sensing, DNA discrimination, and detection of cancer biomarkers [71, 72]. Given the abundance of open sites on layered MOFs, pioneered work by the Lin group described the construction of layered MOFs (NA@Zr-BTB/F/R) and their application in the determination of mitochondrial dysregulation (Fig. 2a) [73]. The layered MOFs with Kagome topology built from {Zr_6_} cluster and 2,4,6-tris(4-carboxyphenyl) aniline ligands were modulated by formic acid through the solvothermal method. The monolayer Zr-BTB is about 1.5 ± 0.1 nm, which is consistent with the height of the formic acid connected {Zr_6_} cluster. Then, the active layer was afforded by post-synthetically modification with GSH-selective (2E)-1-(2′-naphthyl)-3-(4-carboxyphenyl)-2-propen-1-one (NA), pH-sensitive fluorescein isothiocyanate (FITC), and GSH/pH-independent rhodamine-B isothiocyanate (RITC). The fluorescent intensity along with GSH deletions and matrix acidification was reflected by the signals from FITC, RITC, and NA, respectively. As expected, the signals from FITC and RITC were constant, while NA signals increased with the variation of GSH concentrations. In parallel, the constant signals of NA and RITC change signals obviously of FITC when the solution pH changes, which is agreed with the results at the cellular level. More importantly, there is a good linear correlation between the ratiometric fluorescent intensity and the GSH concentrations and pH variations. Mitochondrial dysfunction impacts GSH consumption and hydrogen ion effluxing, which has been implicated in diseases chemoresistance cancers. Thereafter, the layered MOFs enabling ratiometric fluorescent detection for GSH concentrations and pH values have pinpointed the role of mitochondrial DNA alterations on the onset of chemoresistance with extensively practical applications.

Recently, the emergence of antibiotic-resistant bacteria [74, 75] has underscored the urgent need for rapid testing technologies of antimicrobial susceptibility. Thereby, suitable antibiotics could be prescribed in order to provide effective therapeutic options. In comparison with the time-consuming determination, the biosensor can be a suitable strategy for susceptibility testing. Based on this ground, 2D MOFs assisted electrochemiluminescence biosensors (Ru-Con A@ NH_2_-MIL-53(Al)) was developed for the detection of Gram-negative bacteria and antimicrobial susceptibility (Fig. 2b) [39]. The electrogenerated chemiluminescence (ECL) biosensor comprised three components, involving recognition molecule concanavalin A, electrode-modified material NH_2_-MIL-53(Al) nanoplate, and ECL probe ruthenium(II) complex. The ECL signals decreased in proportional to the increasing concentrations *of Escherichia coli (E. coli)* BL21, owing to the specifical recognization by concanavalin A modified MOF material. Strikingly, the detection limit reached up to 16 cells/mL, which is even lower than the CRISPR-Cas9-triggered fluorescence signals amplification. Besides, the Ru-Con A@ NH_2_-MIL-53(Al) with excellent sensitivity, selectivity, and stability was also suitable for rapid detection of antimicrobial susceptibility, which may facilitate antibiotic stewardship in the clinical application.

### Molecular imaging

Molecular imaging with non-invasive characteristics is an important methodology for the identification of disease progression [76, 77]. Contrast agents with good biological imaging performance are urgently needed to meet the requirements in clinical settings [78, 79]. Especially, the resolution of imaging in cancer diagnostics is important for real-time guidance of surgical resection, as well as the early detection of tumors. Fluorescence imaging with high spatial resolutions is an indispensable tool for cancer diagnosis in preclinical research [80, 81]. Biodegradable MOF nanosystems (DNA@Cu-MOF) integrated DNAzyme-assisted fluorescence signal amplification was applied for imaging deregulated miRNA-related hypoxic tumors [82]. In their structure design, the hypoxia-responsive Cu-MOFs were constructed by the surfactant-assisted solvothermal method. When encountering the low oxygen environment in solid tumors, the MOFs degraded and released the loaded Cu-specific DNAzyme precursor. The DNAzyme precursor displaced by the aberrant miRNA resulted in the recovery of fluorescence signals in an expression-dependent manner. Noteworthy, the hypoxia and miRNA are readily applied for initiating sustained signal amplification, fulfilling the requirements for the temporal and spatial detection in vivo.

**Fig. 3:**
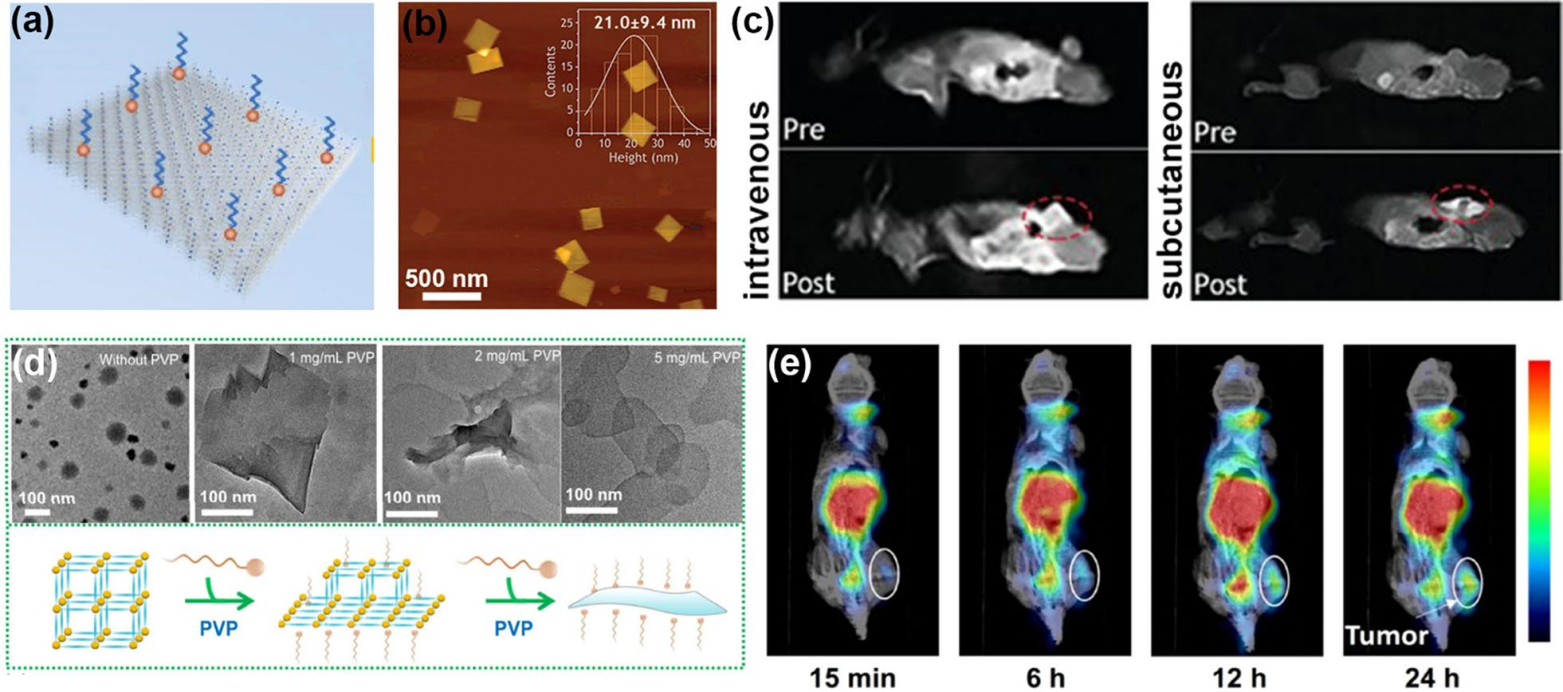
**a** Structural illustration of lanthanide porphyrin MOFs with planar topography. **b** Representative image and the thickness of PPF-Gd measured by atomic force microscope. **c** The MRI application of as-designed PPF-Gd. Reprinted with permission from Ref. [58], Copyright 2019, China Science Press. **d** The Zn-TCPP nanosheets were fabricated by the surfactant-assisted synthesis method. **e** In vivo single-photon emission computer tomography imaging after i.v*.* injection of ^99m^Tc labeled Zn-TCPP@PEG. Reprinted with permission from Ref. [85], Copyright 2019, Springer

However, single imaging modalities often fail to collect reliable clinical information. Currently, combining multiple imaging modalities is more desirable for practical applications [83, 84]. A MOF bridged by porphyrin derivatives and lanthanide elements exhibits outstanding magnetic and luminescent properties, thereby making them promising candidates for diagnostic applications. In this aspect, Xia et al. designed and developed a series of lanthanide porphyrin MOFs (PPF-Ln, Ln = Ce, Eu, Gd, Tb, and Ho) with a surfactant-assisted wet chemistry strategy (Fig. 3a) [58]. The gadolinium-based porphyrin framework (PPF-Gd) integrated TCPP and lanthanide element Gd. The PPF-Gd with a thickness of 21.0 ± 9.4 nm exhibits excellent photoluminescence properties and magnetic resonance imaging capability (Fig. 3b, c). Interestingly, the PPF-Gd exhibited negligible side effects and a biodegradation profile in response to intratumoral acidity. This work further supports the great potential of MOF-based systems with deep tumor penetration and controllable biodegradability, which may broaden the practical application of bimodal imaging technology.

To improve the colloidal stability and biocompatibility, porphyrin-based MOF nanosheets utilizing Zn^2+^ as connecting metal ions were synthesized, and surface modified with polyethylene glycol (PEG) (Fig. 3d) [85]. Interestingly, the as-designed MOF nanosheets are easily labeled with gamma emission radioisotope ^99m^Tc by simple mixing. Thereby, the ^99m^Tc labeled Zn-TCPP@PEG possesses enormous potential for in vivo single-photon emission computer tomography imaging (Fig. 3e). For 4T1-bearing mice, the ^99m^Tc labeled Zn-TCPP@PEG displayed a high degree of tumor homologous ability, and the results were agreed well with the quantification of Zn according to inductively coupled plasma spectrometry. Moreover, these 2D MOFs exhibited indiscernible long-term toxicity, demonstrating the great potential of biodegradable multifunctional nanoplatform for theranostic applications.

### Biomedicine

The porosity MOFs with planar topology provided high surface-to-area, abundance active sites, easily surface modification for applications across biology and medicine, including 3D printing [86, 87], tissue engineering [88, 89], wound healing [90, 91], inflammatory disease [92, 93], or cancer treatment [94, 95]. In this section, recent progress of layered MOFs in antibacterial and/or antitumor application was overviewed, with an emphasis on ROS-based treatment.

### Antibacterial application

**Fig. 4:**
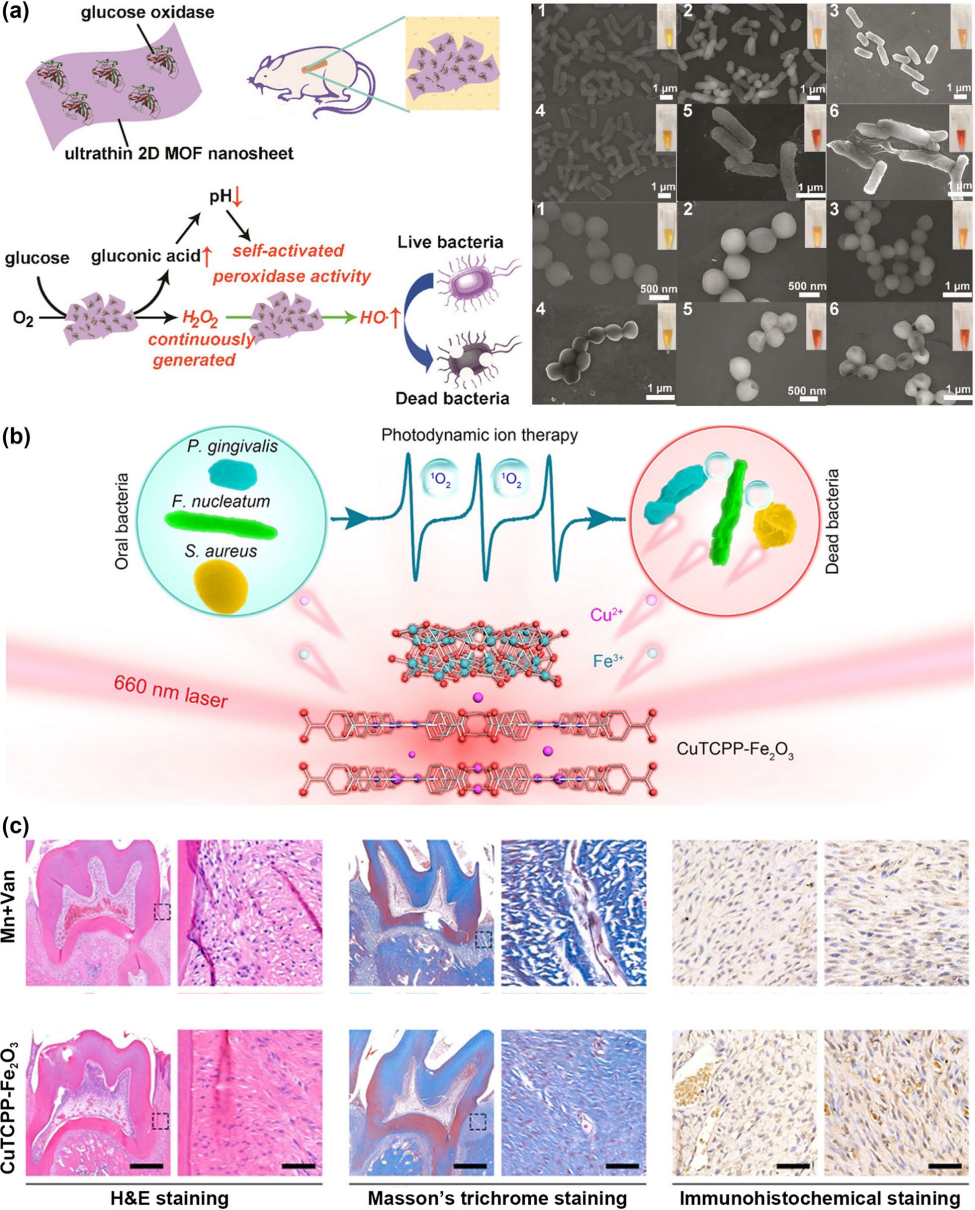
**a** Illustration of the self-activated cascade reaction catalytic by MOF/GOx hybrid nanosheets. Scanning electron microscopic observation of *E. coli* and *S. aureus* after indicated treatment. 1: PBS, 2: glucose, 3: glucose + MOF, 4: MOF/GOx, 5: glucose + GOx, 6: glucose + MOF/GOx, respectively. Reprinted from Ref. [99]. with permission of American Chemical Society, Copyright 2019. **b** Carton representation of the antibacterial mechanism of Cu-TCPP/Fe_2_O_3_ mediated photodynamic ion therapy. **c** Histological analysis of periodontium stained by hematoxylin and eosin (H&E), Masson's trichrome, vascular endothelial growth factor (VEGF), and CD31. Scale bars represent 500 μm for low-magnification, while 50 μm for high-magnification and immunohistochemical images. Reprinted from Ref. [40] with permission of American Chemical Society, Copyright 2021

Chronic diabetic wounds suffer from unexpected epithelium injuries because of the inability to spontaneously heal and vulnerability to bacterial infection [96, 97]. Efficient strategies to promote injury recovery require angiogenesis promotion, collagen deposition, and re-epithelialization simultaneously to combat chronic nonhealing wounds. The emergence of nanotechnology has facilitated wound closure and addressed pathophysiologic abnormalities in patients with diabetes. For example, copper-based MOFs (HKUST-1) displayed improved wound closure by modulating the release of copper ions to stimulate angiogenesis and collagen deposition [98]. Compared with three-dimensional bulk MOFs, 2D MOFs exhibited superior enzymatic catalysis activity due to their unique topological structure. Thereby, ultrathin MOF (Cu-TCPP(Fe)) with superior peroxidase-mimic activity was selected to construct a self-activated cascade nanoreactor for promoting in vivo wound closure (Fig. 4a) [99]. Glucose oxidase (GOx) was physically adsorbed onto Cu-TCPP(Fe) nanosheets, demonstrating the efficient conversion of glucose into gluconic acid and H_2_O_2_. The generated gluconic acid and H_2_O_2_ decreased the environmental pH and offered sufficient substrates, which synergistically activated the peroxidase-like activity of Cu-TCPP(Fe) nanosheets. Overall, the hydroxyl radical generated from MOF-based hybrid nanocatalyst possesses a robust antibacterial effect and negligible damage to normal tissues.

Similarly, ferric oxide-modified porphyrinic MOF layers (Cu-TCPP/Fe_2_O_3_) were employed to treat chronic diseases like periodontitis (Fig. 4b) [40]. The hybrid nanosheets constructed by atomic layer deposition (ALD) were incorporated into a poly(ethylene glycol) matrix to form a photoresponsive ointment. As schematically illustrated, this surface-engineered MOF heterojunction induced efficient killing towards diverse oral pathogens through synergistic ROS production induced by PDT and ion intervention. According to the theoretical calculation, the metal-connected MOF structure facilitated charge transfer and improved light-harvesting capacity, leading to enhanced photocatalytic activity. As a comparison, the photodynamic ion therapy triggered by ALD-engineered MOFs shows a better antimicrobial effect than the clinical antibiotic treatment, like minocycline and vancomycin. These MOF layers exhibit broad-spectrum antimicrobial activity, which may provide a universal synthetic method to optimize periodontitis treatment by reducing inflammation and promoting angiogenesis.

### Cancer treatment

MOF structures possess the advantages of a variety of organic linkers/inorganic built units, infinite arrangements, fine-tuning potential. Compared to the pristine 3D MOF, MOFs with sheet-like morphologies confers their larger surface area, higher flexibility, and more exposed active sites. Owing to these unique features, 2D MOFs are increasingly used in the drug delivery system, especially for oncology treatment (Table 3) [100–106]. Concerning pharmaceutical applications, 2D MOFs often exhibit high drug upload capacity towards various active pharmaceutical ingredients, including chemotherapeutics, antigens, antibodies, and immunoadjuvants. Besides, active pharmaceutical ingredients loaded by planar MOF enable overcoming issues associated with stability, solubility, and systematic cytotoxicity. For example, leaf-shaped MOF (NZIF-L) through rapid scale-up synthesis have been employed for co-delivery of chemotherapeutic DOX and imaging agents 4,4'-(1,2-diphenylvinyl)-1,2-di-(phenyl carboxylic acid) (TCPE) [100].

**Table 3:** Recent breakthroughs associated with 2D MOFs for oncology treatment

2D MOFs	Therapeutics	Drug loading	Treatment	Cells/animals	Refs.
NZIF-L	DOX	~ 3.5 wt% for DOX^a^	Chemotherapy	HeLa cells	[100]
PPF	SOR	84% for SPR^b^	Molecular targeted therapy	McA RH7777-bearing rats (Subcutaneous)	[101]
Ti-TBP	TBP	–	Type I PDT	CT26-bearing BALB/c mice (Subcutaneous)	[102]
Cu-TCPP	Cu^2+^, TCCP, and DOX	33% for DOX^b^	Chemotherapy/PDT/ GSH consumption	4T1 bearing nude mice (Groin)	[103]
Gd-TCPP	Gd^3+^, TCCP, and NC-FAA	–	MRI/SDT/ Starvation therapy/ GSH consumption	A498 bearing mice (Subcutaneous)	[104]
W-TBP	TBP, CpG, and αPD-L1	87.9% for CpG^b^	PDT/Adjuvant/ICB	TUBO bearing mice (Bilateral subcutaneous)	[105]
Hf-TBC	TBC, and IDOi	4.7 wt% for IDOi^a^	PDT/ICB	MC38 bearing mice (Bilateral subcutaneous)	[106]

^a^Drug loading efficiency

^b^Drug loading capacity

**Fig. 5:**
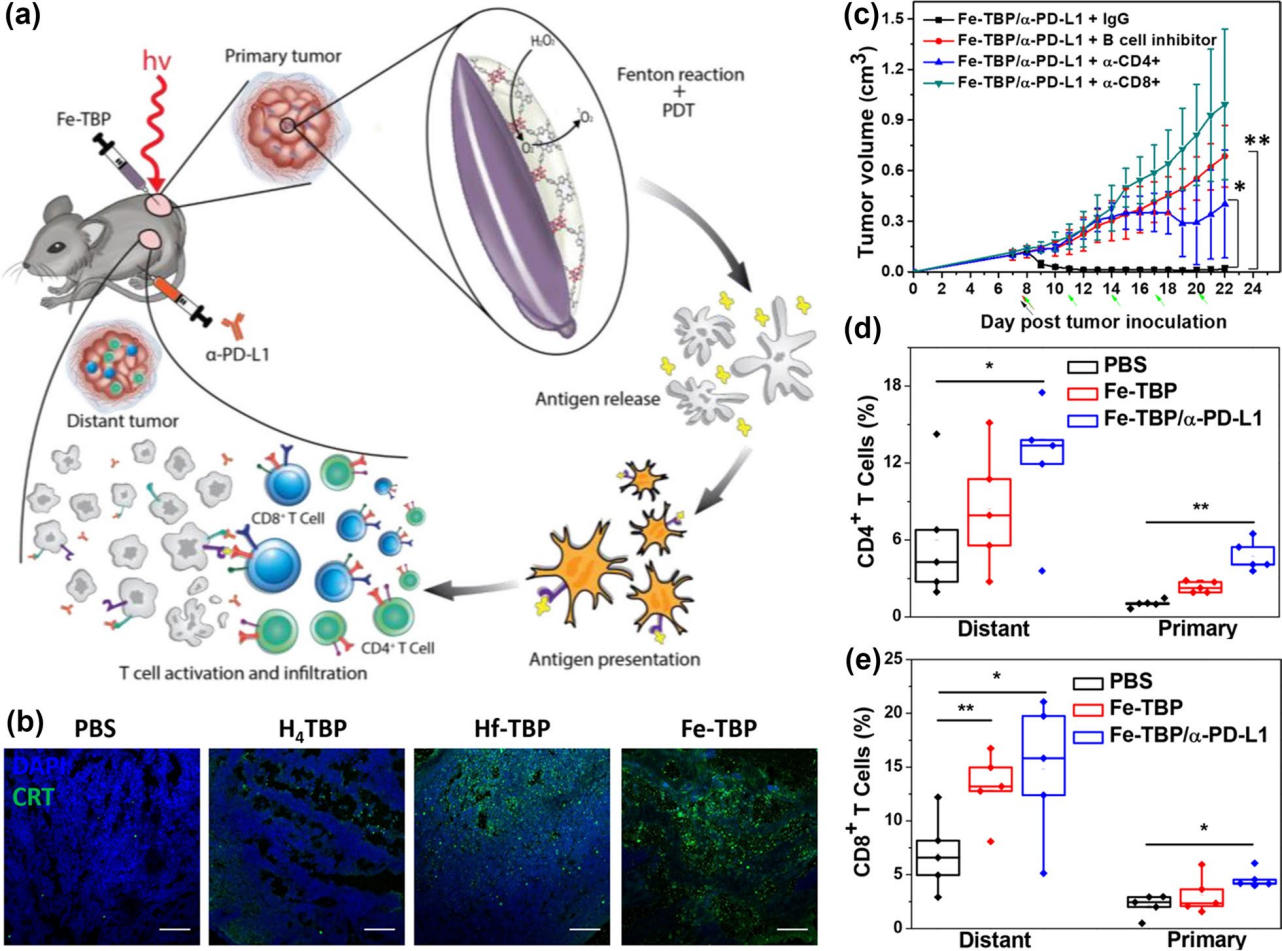
**a** Schematic illustration of using Fe-TBP to overcome hypoxia for PDT primed cancer immunotherapy. **b** Immunofluorescence staining of CRT exposure. **c** Tumor growth curves of CT26 tumor-bearing mice with T cell or B cell depletion.The percentage of tumor-infiltrating (**d**) CD4^+^ T cells, and (**e**) CD8^+^ T cells. Reprinted from Ref. [59] with permission of American Chemical Society, Copyright 2021

MOFs with planar topology possess their unique superiority in ROS generation by nanocatalytic medicine [107, 108]. Generally, the precisely arranged framework enables the diffusion of ROS and molecular oxygen. At the same time, photosensitizers serve as the building block of MOFs that can effectively avoid self-quenching. Pioneered work by Lin’s group has reported a series of layered MOF structures with enhanced PDT performance constructed from 5,10,15,20-tetra(*p*-benzoato)porphyrin (TBP) [102, 105], 5,10,15,20-tetra(*p*-benzoato)chlorin (TBC) [106], and 5,10,15,20-tetra(p-benzoato)bacteriochlorin (TBB) [109]. To potentiate PDT efficacy, iron-oxo clusters-based MOFs (Fe-TBP) were developed to overcome tumor hypoxia by decomposition of endogenous H_2_O_2_ (Fig. 5) [59]. As evidenced by immunofluorescence, the immunogenic PDT elicited by Fe-TBP dramatically improved the calreticulin (CRT) exposure (Fig. 5b). Moreover, the Fe-TBP mediated PDT is capable of elevating the object response of anti-programmed death-ligand 1 (α-PD-L1) treatment. In the colorectal tumor model, necrotic tumor histology was observed after Fe-TBP + α-PD-L1 treatment, demonstrating that the combinatory treatment elicited obvious abscopal effects. The Fe-TBP plus α-PD-L1 group showed a significant increase of tumor-infiltrating CD4^+^ and CD8^+^ T cells in both primary and distant tumors (Fig. 5c). In turn, the immunotherapeutic efficacy was comprised by depletion of either B cells or CD4^+^ or CD8^+^ T cells (Fig. 5d, e). Overall, this study presents a general strategy to alleviate hypoxia in solid tumors for PDT primed immunotherapy of colorectal carcinoma.

**Fig. 6:**
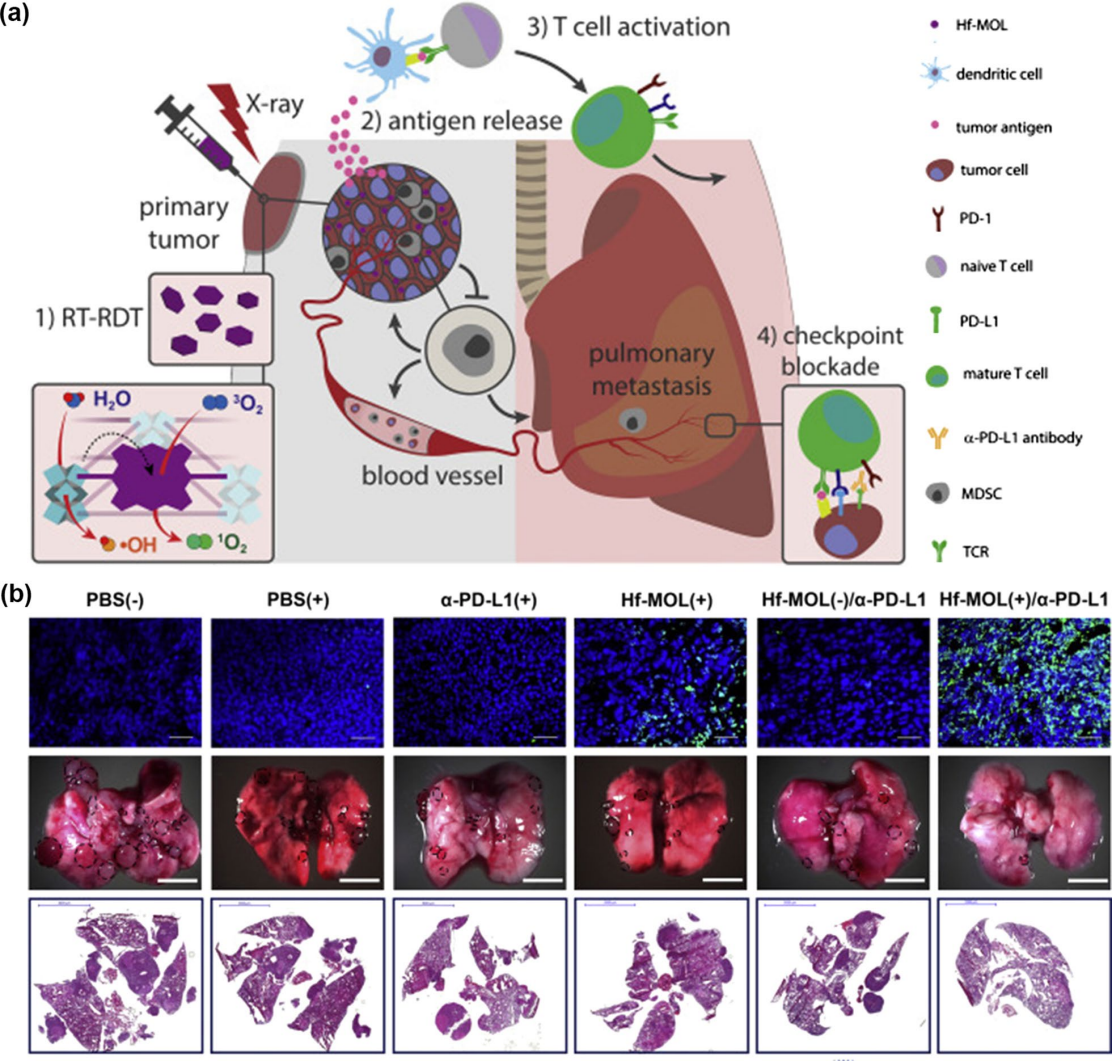
**a** Scheme of Hf-MOL triggered RT-RDT to strengthen checkpoint blockade. **b** Immunofluorescence staining of apoptotic in 4T1 tumor and corresponding lung metastasis results recorded by tumor nodules and H&E staining. Scale bars for terminal deoxynucleotidyltransferase mediated DUTP-biotin nick end labeling (TUNEL) is 100 mm, scale bars represent 5000 mm for images of tumor nodules in the lungs and their histology sections. Reprinted with permission from Ref. [60], Copyright 2019, Cell Press

Despite being approved by Food and Drug Administration, the clinical application of PDT in solid tumors is still limited to superficial malignant lesions. Radiotherapy (RT) overcomes the limitations of tissue penetration but suffers from radioresistance and unwanted side effects [110]. Many innovative kinds of research have been devoted to developing radio enhancers for addressing the safety issues accompanied by using low-dose X-rays [111]. As a paradigm, Ni et al. designed and developed an ultrathin MOF layer (Hf-MOL) for radiotherapeutic enhancement (Fig. 6) [60]. The as-designed Hf-MOL was synthesized by the solvothermal reaction from HfCl_4_ and DBP with acetic acid as modulators. Owing to the reduced dimensionality, Hf-MOL allows rapid diffusion of ROS/O_2_. In the orthotopic breast cancer model, RT-RDT treatment exhibited a superior tumor regression effect even at a radiation dose as low as 1 Gy/fraction. Combination with α-PD-L1 this innovative platform resulted in remarkable antimetastatic effects in histological examination (Fig. 6b), which can be explained by reactivating antitumor immunity and reversing tumor immunosuppression. In addition, this synergy treatment also elicited robust abscopal effects in bilateral colorectal carcinoma, head and neck cancer, and breast cancers, demonstrating a generalized strategy for radioimmunotherapy of advanced malignancies.

## Conclusion and prospect

At present, 2D-MOF materials integrate ultrathin thickness, large surface-to-volume ratio, variety of ligands, permanent porous structure, and highly designability, which has steadily exhibited great potential in nanomedicine and biology. With the development of new nanofabrication technologies, ultrathin MOF nanosheets with desired features will be brought to the forefront of advanced materials to broaden their fundamental and preclinical investigations. Despite remarkable achievements that have been made in the bioapplications of 2D MOFs, this research is still in its primary stage. Shortcomings and deficiencies originating from 2D MOFs, such as the physiological stability and long-term toxicity, continuous industrial production, precise control of lateral size, and lack of innovative applications, still need to be addressed. Even though preliminary research involving 2D MOFs has expanded rapidly, the utilization of 2D MOFs for biomedical applications is approached with cautious optimism owing to the lack of comprehensive material characterization and toxicological studies, especially for those involving long-term administration. Meanwhile, the toxicity of 2D MOFs relies on the synthetic/processing process accompanied by changes in the material composition and degradation characteristics, making it difficult to precisely assess the hazard potential of 2D MOFs. To promote industrial production, considerable efforts were devoted to developing efficacious nanofabrication strategies, integration of emerging technologies such as surface acoustic wave assisted exfoliation, high-throughput droplet microfluidic synthesis, and surfactant-free and scalable general strategy. Furthermore, 2D MOF design should also be focused on the prospect in crystal dissolution-growth kinetics, topotactic transformation, machine-learning for guiding synthetic mythology and control over morphology. Besides, the synthesis in large-scale, high-quality 2D MOFs with superior optical, electrical and magnetic properties is still lacking. In terms of the atomically layered MOF structures, it is necessary to broaden the exploration and promote scientific developments.

In the next generation of nanotechnology, 2D MOFs with more flexibility and tunability should be focused on the development requirements towards wearable devices, 3D printing, and artificial intelligence. Taking the advantage of the flexibility derived from the planar topography, ultrathin MOF nanosheets are ideal alternatives to fabricate wearable and implantable devices for autonomous monitoring of physiological markers. Moreover, it is profoundly meaningful to construct composite materials combining the advantages of each module, thus obtaining hybrid materials with good application prospects, such as transplantable artificial tissues, photosynthetic artificial organelles, and other medical equipment. Overall, the 2D MOFs with high performance and high integration will have good prospects as technologically indispensable materials in the future.

## Data Availability

Yes.

## Abbreviations

MOFs: Metal-organic frameworks
ROS: Reactive oxygen species
PDT: Photodynamic therapy
RDT: Radiodynamic therapy
CDT: Chemodynamic therapy
TCPP: Tetrakis(4-carboxyphenyl)porphyrin
FITC: Fluorescein isothiocyanate
RITC: Rhodamine-B isothiocyanate
ECL: Electrogenerated chemiluminescence
PEG: Polyethylene glycol
Gox: Glucose oxidase
HKUST-1: Copper-based MOFs
ALD: Atomic layer deposition
H&E: Hematoxylin and eosin
VEGF: Vascular endothelial growth factor
NZIF-L: Leaf-shaped MOF
TCPE: 4,4′-(1,2-Diphenylvinyl)-1,2-di-(phenyl carboxylic acid)
TBP: Tetra(*p*-benzoato)porphyrin
TBC: Tetra(*p*-benzoato)chlorin
TBB: Tetra(*p*-benzoato)bacteriochlorin
CRT: Calreticulin
α-PD-L1: Anti-programmed death-ligand 1
RT: Radiotherapy
TUNEL: DUTP-biotin nick end labeling
